# Potential Lactic Acid Bacteria Characterized From the Gastrointestinal Tract of Butter Catfish, *Ompok pabda* for Implying as Probiotics in Aquaculture

**DOI:** 10.1155/ijm/8825568

**Published:** 2026-06-01

**Authors:** Rafiatul Jannat Rifa, Md. Sakhawat Hossain, Chandrika Mondal, Md Imtiaz Ahamed, Khadiza Tul Kubra, Bushra Bente Rahman Prapti, Mohammad Matiur Rahman, Tanvir Rahman, Md. Rafiqul Islam Sarder, Mariom

**Affiliations:** ^1^ Department of Fisheries Biology and Genetics, Bangladesh Agricultural University, Mymensingh, Bangladesh, bau.edu.bd; ^2^ Department of Aquaculture, Bangladesh Agricultural University, Mymensingh, Bangladesh, bau.edu.bd; ^3^ Department of Microbiology and Hygiene, Bangladesh Agricultural University, Mymensingh, Bangladesh, bau.edu.bd

**Keywords:** aquaculture, lactic acid bacteria, *Ompok pabda*, probiotic potential, *Weissella cibaria* OPL3, *Weissella confusa* OPL2

## Abstract

Probiotics derived from an identical host′s gastrointestinal (GI) system confer superior colonization and provide greater benefits than other commercial sources. This study reports on the probiotic potentials of lactic acid bacteria (LAB) identified from the gut content of *Ompok pabda* through morphological, physiological, and molecular sequencing for their usefulness in aquaculture. Molecular sequencing confirmed two LAB strains affiliated with the genus *Weissella;* namely *Weissella confusa* OPL2 and *Weissella cibaria* OPL3. Each strain was evaluated for its capability to withstand conditions relevant to feed application and gastrointestinal survival, including fluctuations in temperature, NaCl concentration, pH, and gut bile. Both strains consistently tolerated acidic pH (pH 2–5), bile concentration up to 7.5%, elevated temperature (4°C–45°C), and salt concentration (3%–6%). In addition, the strains demonstrated strong cell‐surface properties, including higher hydrophobicity with time (68.77 ± 0.17*%* to 69.36 ± 0.41*%* in xylene, 65.62 ± 0.48*%* to 67.73 ± 0.36*%* in toluene), pronounced autoaggregation capacity (64.15 ± 0.35*%* and 64.12 ± 0.53*%*), notable coaggregation rates of 52.79 ± 2.15*%* and 53.05 ± 0.35*%* with *Bacillus aerius*, and 50.11 ± 0.16*%* and 49.51 ± 0.81*%* with *Staphylococcus epidermidis*, for *W. cibaria* OPL3 and *W. confusa* OPL2, respectively. Furthermore, the strains showed marked in vitro antagonism against *Aeromonas veronii* OPP8 and *Plesiomonas shigelloides* OPP9. Safety assessment further supported their suitability as probiotics, as they were found nonhemolytic in nature, as well as susceptible to several commonly used antibiotics. Therefore, the findings indicate *W. cibaria* OPL3 and *W. confusa* OPL2 as a safe and promising probiotic candidate from *O. pabda* with clear probiotic potentials in the aquaculture industry.

## 1. Introduction

Aquaculture has emerged as a vital industry for addressing the escalating global demand for high‐quality protein, particularly in freshwater environments [1–3]. As one of the leading fish‐producing countries, Bangladesh ranked fifth in aquaculture production according to the FAO [4]. About 12% of the population depends on fisheries for their livelihood, with aquaculture contributing 2.53% to the national GDP and accounting for 60% of the country′s animal protein intake in Bangladesh [5]. Aquaculture contributes to the annual fish production of 5.02 MMT, which represents 2.57% of Bangladesh′s total inland fish production [5].

Among the commercial indigenous species, the freshwater catfish, *Ompok pabda* (often called “pabda” or “butter catfish”) is distinguished by its high market value, delicate texture, and favorable nutritional characteristics [6, 7]. This species contains a higher level of crude protein (27%), crude lipid (3.6%), and ash content (5.3%) [6], compared with other fish species [8], thus increasingly recognized in Bangladesh as well as other Southeast Asian countries for its potential in commercial aquaculture. The culture of this species is now being intensified and well‐practiced all over Bangladesh. However, fish farmers are facing considerable challenges such as disease outbreaks, heavy mortalities due to microbial infections, lower growth rate and productivity, and ultimately great economic loss [9, 10]. To control infectious diseases and microbial infections, the most commonly used measure is the application of antibiotics, therapeutics, and other treatments [11]. The uncontrolled and indiscriminate use of these therapeutics leads to serious problems in fish farming, including the spread of antibiotic resistance, environmental risk, and human health issues [2]. In addition, the profitability of the aquaculture sector is also being diminished by feed cost, which makes up more than 70% of the total production cost [12]. Therefore, fish farmers must adopt alternative disease management practices that are sustainable as well as eco‐friendly to reduce the ongoing problems. The incorporation of probiotics, particularly lactic acid bacteria, is regarded as the most sustainable approach in aquaculture systems [2, 13, 14].

Probiotics are live microorganisms, usually bacteria or yeasts, that provide health benefits by maintaining a healthy balance of microbes in the digestive system when consumed in adequate amounts [15–17]. Among probiotics, LAB are widely recognized and defined as Gram‐positive, nonmotile, nonspore‐forming bacteria that produce lactic acid during fermentation [16], and include the genera *Lactobacillus, Lactococcus, Streptococcus, Vagococcus, Weissella, Pediococcus, Enterococcus*, and *Carnobacterium*. LAB are considered the most promising probiotics due to their significant role in immune system modulation, antimicrobial action against pathogens, prevention of infections, and intestinal microbial balance [15, 18–22]. They have superior adaptability to the host environment, better gut colonization ability, and formation of a barrier against pathogens; these attributes make them preeminent over other probiotics [13, 23].

LAB strains that are sourced from host gut elicit better probiotic functions compared with nonnative strains. Numerous research has been conducted on the isolation of probiotics from fish species like Gangetic mystus (*Mystus cavasius*) [24], Stinging catfish (*Heteropneustes fossilis*) [25], Nile tilapia (*Oreochromis niloticus*) [14], crayfish (*Procambarus clarkia*) [26], freshwater prawn (*Macrobrachium rosenbergii*) [27], Turbot fish (*Scophthalmus maximus*) [28], leaving a gap in knowledge regarding the gut microbiota of *O. pabda*. To our knowledge, there has been no study pertaining to the identification of probiotic LAB from the gut content of the commercially important catfish, *O. pabda,* so far. Considering the importance of this species in the national economy and to minimize problems associated with intensive culture practice, this research is aimed at isolating, identifying, and characterizing the potential LAB strains from *O. pabda* for their probiotic properties to be addressed as the growing need for species‐specific probiotic solutions. The indigenous LAB from the gastrointestinal tract of *O. pabda* could provide better colonization, improved host compatibility, and strong disease resistance compared with nonhost‐derived probiotic strains.

## 2. Materials and Methods

### 2.1. Collection of Fish Sample

Twelve live and healthy *O. pabda* fish with a mean length of 18.275 ± 1.76 cm and weight of 40.03 ± 3.67 g were acquired from three different sources in Mymensingh namely Satata Matsha Prajanan Kendra and Fishery, Sabir Agro Fish Farm, and Sheshmor fish market. The study was carried out at Bangladesh Agricultural University (BAU), Mymensingh (Latitude 24.72291° N, Longitude 90.43077° E), Bangladesh. After collecting, the fish sample was conditioned in a cistern for 2 days to evacuate the gut and then moved to the laboratory for bacteria isolation. Conditioning was performed so that unwanted pathogenic bacterial load reduces over time [25]. Additionally, it would help to stabilize and enrich indigenous microbiota, especially LAB.

### 2.2. Isolation of Lactic Acid Bacteria

**Figure 1:**
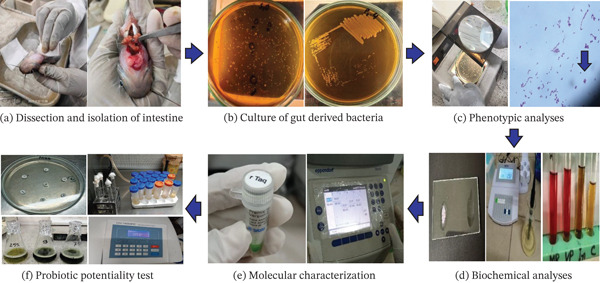
Isolation, characterization, and identification of lactic acid bacteria from the gut of *O. pabda.* (a) Dissection and isolation of intestine. (b) Culture of gut‐derived bacteria. (c) Phenotypic analyses. (d) Biochemical analyses. (e) Molecular characterization. (f) Probiotic potentiality test.

At first, the fish samples were rubbed with a solution of 70% alcohol to avoid cross‐contamination and anesthetized with a 60 mg/L solution of MS 222 [29]. After that, the fish were dissected and the intestines were collected aseptically by using sterile forceps (Figure 1a). For microbial isolation, about 0.5 g intestine was taken from each sample and homogenized with 5000 *μ*L of sterile 0.87% (*w*/*v*) physiological saline. This ratio was maintained based on 1000 *μ*L physiological saline for every 0.1 g of intestinal tissue. During crushing, the homogenizer was placed on an iced water‐filled basket so that the heat generated during crushing could not kill the intestinal microbes. Then the homogenate solution was moved into a sterile test tube and 100 *μ*L of the solution was spread on de Man Rogosa Sharpe (MRS) agar (Figure 1b) and incubated at 37°C for 24–72 h.

### 2.3. Morphological and Biochemical Characterization of LAB

The selected isolates which are presumed to be lactic acid bacteria were characterized based on colony characteristics (colony shape, pigmentation, elevation, edge and arrangements) and other preliminary tests (growth on selective agar medium, mobility, Gram staining and aerobic–anaerobic testing) (Figure 1c). After morphological screening, several biochemical analyses, including catalase activity, indole test, methyl red test, Voges–Proskauer test, acid formation test, and carbohydrate fermentation test were carried out (Figure 1d).

#### 2.3.1. Growth on Selective Culture Media

Lactic acid bacteria grow on different selective media due to their specific nutritional requirements and growth characteristics. Selective media can be formulated to provide these nutrients to LAB while inhibiting the growth of non‐LAB. The isolates were cultured on nutrient agar (NA), Trypton soya agar (TSA), and MRS agar, a selective medium for LAB. NA and TSA were used initially to provide a nonselective environment for the growth of a wide range of bacteria, allowing for subsequent differentiation of LAB using MRS agar, which is the selective media for LAB propagation.

#### 2.3.2. Gram Staining, Mobility and Aerobic–Anaerobic Tests

The isolates were subjected to Gram staining and tested for aerobic–anaerobic characteristics following the methods described by Schillinger and Lucke [30] and Dykes et al. [31], respectively. The motility test was performed by the stab culture method [32]. After inoculation, the culture system was kept at 37°C for 24–48 h. After incubation, the medium was observed to examine whether the bacterial growth diffused away from the stab line.

#### 2.3.3. Catalase Test

The bacterial isolates were tested by slide catalase test procedure [33]. Initially, a small sample of bacteria was subjected to a clean, sterilized slide using an inoculating loop. After that, 2–3 drops of 3% H_2_O_2_ were added to the colony on the glass slide. If oxygen (O_2_) bubbles are observed immediately after placing H_2_O_2,_ the result is counted as catalase positive.

#### 2.3.4. Indole, Methyl Red, Voges–Proskauer and Acid Formation Test

Gram‐positive, nonmotile, and catalase negative isolates were selected and further screened by biochemical tests, including indole production test, methyl red test, Voges–Proskauer test, and acid formation test [34].

#### 2.3.5. Carbohydrate Fermentation Test

Carbohydrate fermentation properties of the isolates were determined as described by Müller [35] and Bulut et al. [36]. The five basic sugars were used to assess their ability in fermentation, namely sucrose, lactose, maltose, mannitol, and dextrose.

### 2.4. Molecular Identification of LAB

**Figure 2:**
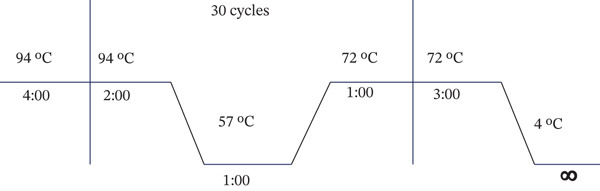
Thermal condition of PCR amplification.

The bacterial genomic DNA was extracted by following the traditional phenol‐chloroform method of DNA extraction [37] and quantified using a spectrophotometer (Jenway Genova, France). Following extraction, the bacterial 16S rRNA gene was amplified by conventional PCR using a thermocycler (Eppendorf AG 22331, Hamburg, Germany) with universal 16S ribosomal RNA gene primers, 27F (5 ^′^‐AGA GTT TGA TCC TGG CTC AG‐3 ^′^) and 1492R (3 ^′^‐TAC GGT TAC CTT GTT ACG ACT‐5 ^′^) (Figure 1e). Each sample had a total volume of 30 *μ*L reaction mixture, which contained 15 *μ*L of the Taq. master (Premix Taq, Version 2.0 with dye, TAKARA Bio, India), 1.5 *μ*L of each primer (forward and reverse primer, conc. 10 *μ*M), 10 *μ*L of nuclease‐free water, and 2 *μ*L of template DNA (about 100 ng). The condition of the thermal cycler is provided in Figure 2. Agarose gel electrophoresis (1%) was used to examine the PCR results and observed the target bands. Two *μ*L of PCR sample was loaded along with 100 bp plus DNA marker (Addbio, Korea) to compare the fragments of amplified PCR product. Amplification of nearly 1450 bp consensual fragment was verified by electrophoresis on 1% TAE agarose gel.

### 2.5. DNA Base Sequencing and Identification of Bacterial Species

The most intense PCR samples were sent for sequencing to the NIB (National Institute of Biotechnology), Bangladesh. Then, the sequence of each isolate was compared with the publicly available reference sequences in the NCBI (National Centre for Biotechnology Information) GenBank (https://www.ncbi.nlm.nih.gov/) by using the Basic Local Alignment Search Tool for nucleotides (BLASTNs) search algorithm with the nonenvironmental sequence option selected [38]. All the obtained sequences were deposited in NCBI GenBank and the accession numbers were acquired.

### 2.6. Phylogenetic Analysis

Sequences with homologs greater than 97% were selected as identified bacteria. Using the closely related species that were retrieved from GenBank, phylogenetic analysis was constructed by Molecular Evolutionary Genetic Analysis (MEGA 11) using the neighbor‐joining method (Maximum likelihood of bootstrap value = 1000).

### 2.7. In Vitro Characterization of Probiotic Properties of LAB Isolates

#### 2.7.1. Tolerance to Different Temperatures and Salt Concentration

The identified LAB strains were incubated at varying temperature (4°C–45°C) to assess their tolerance to cold and elevated temperature, and the growth was observed after 24 h. Similarly, the salt tolerance test was done at two different concentrations of salt (3% and 6%) on TSA media. The culture plates were incubated for 24–36 h. After 36 h, the growth was observed in both concentrations.

#### 2.7.2. Antibiotic Sensitivity Test

The antibiotic susceptibility test was performed by the Kirby–Bauer disk diffusion method [39] using Mueller–Hinton agar (MHA) media. Ampicillin (10 *μ*g), chloramphenicol (30 *μ*g), cefotaxime (30 *μ*g), vancomycin (30 *μ*g), erythromycin (15 *μ*g), tetracycline (30 *μ*g), and ciprofloxacin (5 *μ*g) were used for the studies (Figure 1f). The antibiotic discs (Thermo Scientific Oxoid) were purchased from Oxoid Limited (United Kingdom), and the antibiotics tested were selected based on the recommendations of the European Food Safety Authority (EFSA). The sensitivity of LAB to antibiotics was interpreted based on inhibitory zones (mm) around the bacterial colonies according to guidelines of the Clinical and Laboratory Standard Institute (CLSI).

#### 2.7.3. Tolerance to Low pH

The tolerance of LAB isolates to low pH was assessed following the method of Patel et al. [39], with minor modifications. MRS broth media was adjusted to pH values of 2, 3, and 5 by adding 1% hydrochloric acid (HCL) (Figure 1f). LAB strains were inoculated into the media and incubated at 25°C in a shaker incubator (JSOS‐500 JSR) set to 100 rpm. Then bacterial propagation was measured by recording optical density (OD) at 600 nm using a spectrophotometer (VWR UV 1600PC, Europe).

#### 2.7.4. Tolerance to Gut Bile

The identified LAB strains were examined for bile tolerance using a modified method from Patel et al. [39]. Crude bile was extracted from the gall bladder of *O. pabda* by using a sterile syringe. The bile tolerance of the identified LAB strains was investigated by inoculating overnight cultures into TSB containing 2.5%, 5.0%, and 7.5% gut bile and incubated at 25°C in a shaker incubator (JSOS‐500 JSR) set to 100 rpm (Figure 1f). The OD at 600 nm was recorded at 3, 6, and 9 h of incubation in order to evaluate bacterial growth.

#### 2.7.5. Auto Aggregation Properties

Autoaggregation abilities were investigated according to the procedure described by Collado et al. [40], with slight modifications. The percentage (%) autoaggregation was computed using the equation:

Autoaggregation%=1−AtA0×100%

Where *A*_*t*_ denotes the absorbance of the mixture at time t (2, 4 and 6 h), and *A*_0_ is the absorbance at the starting point.

#### 2.7.6. Coaggregation Assays

Coaggregation of identified LAB strains with *Staphylococcus epidermidis* OPL5 (PQ871368) and *Bacillus aerius* OP2 (PQ871370) was performed following the method described by Fonseca et al. [41]. The opportunistic pathogenic bacteria, *S. epidermidis* OPL5 and *B. aerius* OP2, previously isolated from the gut content of *O. pabda* were used. The coaggregation percentage was determined using the equation:

Coaggregation%=A0−AtAt×100

Where *A*_0_ is the absorbance at the starting point and *A*_*t*_ is the absorbance at time t.

#### 2.7.7. Cell Surface Hydrophobicity Properties

The cell surface hydrophobicity of potential LAB strains was assessed by measuring microbial affinity to xylene and toluene by following the procedure outlined by Yasmin et al. [42] with minor modification. Each assay was performed three times using biological replicates, and data are expressed as follows:

Cell surface hydrophobicity%=A0−AtAt×100

Where *A*_0_ is the initial absorbance before adding the solvents and *A*_*t*_ is the absorbance recorded after 6 h of exposure to xylene and toluene.

#### 2.7.8. Hemolytic Activity

Hemolytic activity was evaluated using 7% sheep blood agar plates, as outlined by Petjul et al. [43]. The resulting hemolytic patterns were classified as *α*‐hemolysis (green zone), *β*‐hemolysis (clear zone), or *γ*‐hemolysis (no clear zone).

#### 2.7.9. Antimicrobial Activity Test

The identified LAB strains were performed for antimicrobial activity by the modified well diffusion agar method as described by Thi et al. [44]. Initially, two indicator pathogenic bacteria, namely *Aeromonas veronii* OPP8 (PQ871381) and *Plesiomonas shigelloides* OPP9 (PQ871382), previously identified from the intestine of *O. pabda* were used. The pathogenic bacteria were inoculated on MHA plates, and wells were aseptically punched into the agar. Then, 100 *μ*L of cell‐free supernatant was poured into the well. Finally, the plates were incubated at 37°C for 24 h, following which the inhibition zones were noted for statistical analysis.

### 2.8. Data Analysis

Every experiment was carried out in triplicate, and the means ± standard deviations of independent copies were used to present the result. The collected data were evaluated using one‐way ANOVA (analysis of variance) followed by Tukey′s HSD (honestly significant difference) post hoc analysis through SPSS (statistical package for the social sciences) software (https://www.ibm.com/products/spss-statistics), and OriginPro (Version 2026; 10.3) software (https://www.originlab.com/), along with statistical significance level of *p* < 0.05.

## 3. Results

### 3.1. Bacterial Isolation and Initial Characterization

A total of 12 bacterial isolates were successfully obtained as pure culture from the intestine of *O. pabda*, and selected for the identification of presumptive LAB based on morphological and physiological traits.

### 3.2. Morphological and Biochemical Screening of LAB

Out of the 12 bacterial isolates, 42% (5/12) were circular in colony shape and 58% (7/12) were irregular, and all the isolates showed entire margins. In terms of cell arrangement, 50% (6/12) were cocci and 50% (6/12) were rods as shown in Table 1. Among the 12 isolates analyzed, 33% (4/12) were Gram‐positive and exhibited facultative anaerobic properties (Table 2). In terms of the motility test, 42% (5/12) were nonmotile, whereas 58% (7/12) were motile. All the isolates were mesophilic in nature (Table 2).

**Table 1:** Colony morphology of the selected bacterial isolates.

Strain names	Shape	Surface	Elevation	Arrangement	Margin	Pigmentation
OPL1	Circular	Smooth	Convex	Chained cocci	Entire	Off white
OPL2	Circular	Smooth	Convex	Chained cocci	Entire	Off white
OPL3	Circular	Smooth	Convex	Chained cocci	Entire	White
OP5	Circular	Smooth	Flat	Clustered cocci	Entire	White
OPL5	Circular	Smooth	Flat	Clustered cocci	Entire	Off white
OP6	Irregular	Rough	Slightly raised	Rod	Entire	Creamy
OP7	Irregular	Rough	Flat	Rod	Entire	Creamy
OP8	Irregular	Smooth	Slightly raised	Rod	Entire	Creamy
OP9	Irregular	Smooth	Slightly raised	Rod	Entire	Yellowish
OP10	Irregular	Smooth	Flat	Rod	Entire	Yellowish
OP11	Irregular	Rough	Flat	Rod	Entire	White
OP12	Irregular	Rough	Flat	Clustered cocci	Entire	White

**Table 2:** Phenotypic characteristics of the selected isolates collected from the intestines of *O. pabda.*

Strain names	Parameters			
	Growth on selective MRS media	Gram staining	Motility	Oxygen requirement
OPL1	−	−	Nonmotile	Aerobic
OPL2	+	+	Nonmotile	Facultative anaerobe
OPL3	+	+	Nonmotile	Facultative anaerobe
OP5	−	+	Nonmotile	Facultative anaerobe
OPL5	+	+	Nonmotile	Facultative anaerobe
OP6	−	−	Motile	Aerobic
OP7	−	−	Motile	Aerobic
OP8	−	−	Motile	Aerobic
OP9	−	−	Motile	Aerobic
OP10	−	−	Motile	Aerobic
OP11	−	−	Motile	Aerobic
OP12	−	−	Motile	Aerobic

*Note:* The symbols “+” denote positive reaction and “−” denote negative reaction; all isolates were mesophilic in nature.

Based on morphological attributes, seven isolates were excluded that exhibited irregular colony morphology, Gram‐negative reaction, and aerobic oxygen requirement properties. The remaining five isolates were considered as presumptive LAB and analyzed for biochemical characterization. The result exposed that out of five isolates, two isolates (40%) showed catalase positive, five isolates (100%) showed indole negative, two (40%) were methyl red negative, and four isolates excluding OPL1 produced acid (Table 3). In addition, these isolates were capable of fermenting a range of sugars such as sucrose (4/5), lactose (2/5), maltose (4/5), mannitol (1/5), and dextrose (5/5) (Table 3).

**Table 3:** Biochemical characteristics of the presumptive LAB isolates obtained from the intestines of *O. pabda.*

Test	OPL1	OPL2	OPL3	OP5	OPL5
Catalase	+	−	−	−	+
Indole	−	−	−	−	−
Methyl red	−	+	+	+	−
Voges–Proskauer	−	−	−	−	−
Acid formation	−	+	+	+	+

Carbohydrate fermentation					
Sucrose	−	+	+	+	+
Lactose	−	+	+	−	−
Maltose	−	+	+	+	+
Mannitol	−	−	−	−	+
Dextrose	+	+	+	+	+

*Note:* The symbols “+” denotes well growth; “−” no growth; OPL1, OPL2, OPL3, OP5, and OPL5: bacterial isolates.

### 3.3. Identification of LAB Through Molecular Approach

**Figure 3:**
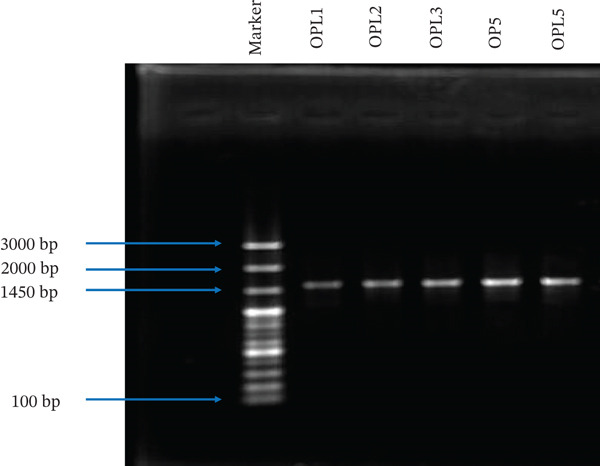
Confirmation of 16S rRNA gene amplification by agarose gel electrophoresis. OPL1, OPL2, OPL3, OP5, and OPL5: bacterial isolates; bp: base pair.

Following the biochemical analyses, the five isolates (OPL1, OPL2, OPL3, OP5, and OPL5) exhibiting LAB‐like characteristics were selected for molecular analysis. Genomic DNA was extracted from the presumptive LAB, and PCR amplification of the target region, 16 s rRNA gene prompted approximately 1450 bp fragments (Figure 3). For precise identification of the test isolates, 16S rRNA gene sequencing followed by sequence analysis using the NCBI‐BLAST software revealed that two isolates were classified as lactic acid bacteria, specifically *Weissella confusa* OPL2 and *W. cibaria* OPL3 (Table 4). The remaining three isolates taxonomically belonged to the genera *Kurthia*, *Staphylococcus*, and *Acinetobacter*. The BLAST results, which exhibited the highest sequence identity and query coverage, facilitated the identification of the closest phylogenetic relatives of each isolate. All the sequences have been deposited in the NCBI GenBank (SUB15008191), and the corresponding GenBank accession numbers for these sequences are provided in Table 4. Among five identified strains, two LAB strains were subjected to further probiotic attributes analysis, whereas three non‐LAB strains were excluded from the evaluation.

**Table 4:** The genomic identity of the sequenced bacterial strains with their GenBank accession number.

Isolates	Sequence length (bp)	Identified strains	% query	Accession number	Closest reference sequence (accession number)
OPL1	965	*Acinetobacter haemolyticus* strain OPL1	100	PQ871364	JX845720.1
OPL2	946	*Weissella confusa* strain OPL2	100	PQ871365	PV270803.1
OPL3	969	*Weissella cibaria* strain OPL3	100	PQ871366	OM736110.1
OP5	946	*Kurthia gibsonii* strain OP5	100	PQ871373	PP389410.1

OPL5	953	*Staphylococcus epidermidis* strain OPL5	100	PQ871368	OP852797.1

### 3.4. Phylogenetic Analysis

**Figure 4:**
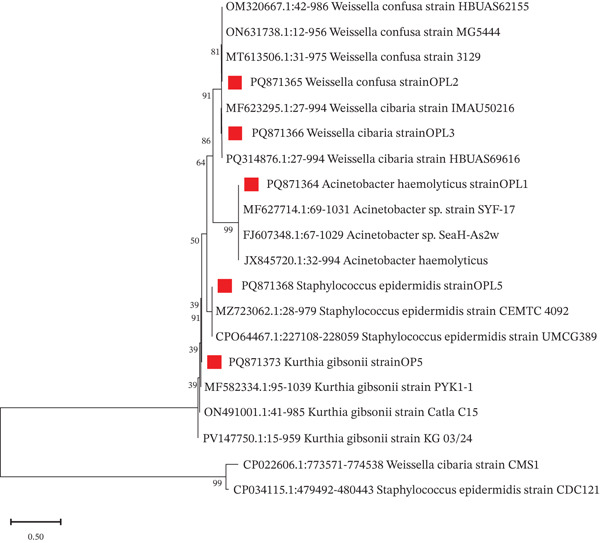
Phylogenetic tree of the identified bacterial strains through 16S rRNA gene sequencing with their respective type strains. (The branching points indicate the bootstrap values; the red square indicates the strains identified from this study).

The phylogenetic analysis illustrated the clustered mechanism of closely related species (Figure 4). *W. confusa* strain OPL2 (PQ871365) and *W. cibaria* strain OPL3 (PQ871366) clustered closely with other strains of this species, such as HBUAS62155, MG5444, 3129, HBUAS69616, and IMAU50216. Similarly, *Acinetobacter haemolyticus* strain OPL1 (PQ871364) was grouped with SYF‐17 and *A. haemolyticus*, indicating evolutionary proximity within the *Acinetobacter* genus with moderate bootstrap support (58). *S. epidermidis* strain OPL5 (PQ871368) was closely related to strains CEMTC 4092 and UMCG389, and *Kurthia gibsonii* strain OPL5 (PQ871373) clustered with strains PYK1‐1, Catla C15, and KG 03/24, emphasizing its evolutionary ties within the *Kurthia* genus.

### 3.5. Probiotic Properties of LAB

#### 3.5.1. Growth on Different Temperature and Salt Concentration

**Figure 5:**
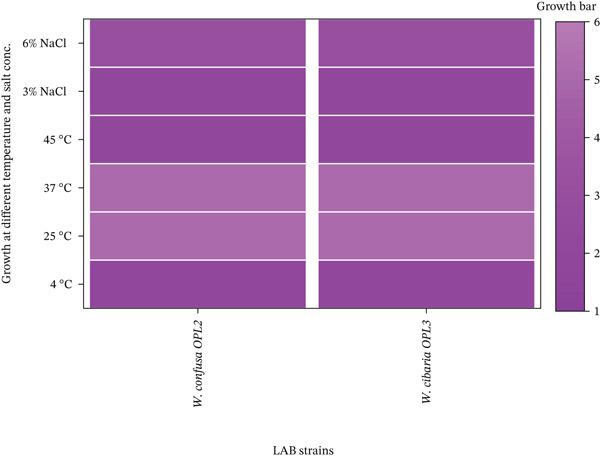
Heatmap showing the growth patterns of LAB strains under different temperatures and salt concentrations. Growth score ranging from 1 to 6, where a higher score indicates more substantial growth (6 represents the highest growth score).

The identified LAB, *W. confusa* strain OPL2 and *W. cibaria* strain OPL3 were further evaluated for their probiotic potentials. The results showed that both *W. confusa* strain OPL2 and *W. cibaria* strain OPL3 were able to grow at 3% and 6% NaCl (Figure 5). Furthermore, both strains exhibited notable tolerance to variable temperatures of 25°C and 37°C. These findings revealed their adaptability to moderate saline and temperature stress conditions.

#### 3.5.2. Antibiotic Sensitivity Profile

**Figure 6:**
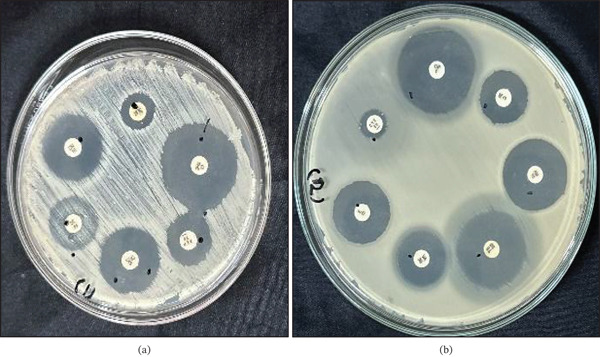
Antibiotic sensitivity pattern of identified LAB strains by agar disc diffusion method; (a) inhibition zone produced by different antibiotic disc against *W. confusa* OPL2, and (b) inhibition zone produced by different antibiotic disc against *W. cibaria* OPL3. Clear zones surrounding the disc reflect susceptibility of the strains to respective antibiotic, whereas absence or reduced zone indicates resistance or low sensitivity. The diameter of each inhibition zone was measured in millimeters and interpreted according to standard guidelines.

The antibiotic sensitivity of identified LAB to seven commonly used antibiotics is presented in Table 5. Bacterial isolates were categorized based on their inhibition zone as follows: resistant (≤ 13 mm), intermediate (14–18 mm), and susceptible (≥ 19 mm) [45]. The test results showed that *W. cibaria* OPL3 showed resistance only against cefotaxime (Figure 6), whereas *W. confusa* OPL2 was resistant against erythromycin (Table 5).

**Table 5:** Antibiogram profile of the identified LAB from the gut content of *O. pabda.*

Strains	Antibiotic discs						
	AM	CTX	CIP	C	TE	E	VA
*W. confusa* OPL2	S	S	S	S	S	R	S
*W. cibaria* OPL3	S	R	S	S	S	S	S

Abbreviations: AM, ampicillin; C, cloramphenicol; CIP, ciprofloxacin; CTX, cefotaxime; E, Erythromycin; R, resistant; S, sensitive; TE, tertracyline; VA, vancomycin.

#### 3.5.3. pH Tolerance

**Figure 7:**
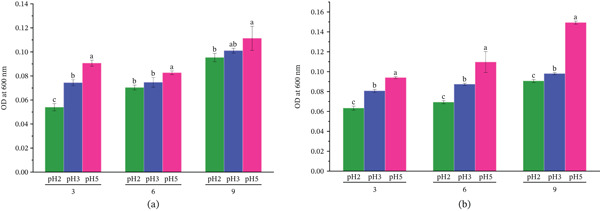
Growth of (a) *W. confusa* OPL2 and (b) *W. cibaria* OPL3 at different pH (2, 3, and 5) over time (3, 6, and 9 h). Paired comparison using Tukey′s honestly significant difference (HSD) test showed significant differences among growth. Each bar represents the value as mean ± standard deviation (*n* = 3). a, b, and c indicate the statistically significant differences; *p* < 0.05. For both strains, growth progressively increased with incubation time, with comparatively higher growth observed at pH 5 than at pH 2 and pH 3.

The results revealed that the LAB strains could withstand the acidic pH. The bacterial propagation was assessed by measuring the OD values at 600 nm. Both LAB isolates grew best at pH values close to neutrality (pH 5). However, statistically significant differences (*p* < 0.05) were observed between treatments (Figure 7). Specifically, one‐way ANOVA revealed a statistically significant effect of pH on bacterial growth for both strains. Post hoc comparisons further confirmed that the OD values at pH 5 were significantly higher (*p* < 0.05) than those at pH 3 and pH 2, indicating that extreme acidity suppressed but did not eliminate the bacterial growth. Among the two strains, *W. confusa* strain OPL2 exhibited significantly (*p* < 0.05) higher growth at pH 2 compared with *W. cibaria* strain OPL3 (Figure 7).

#### 3.5.4. Bile Tolerance

**Figure 8:**
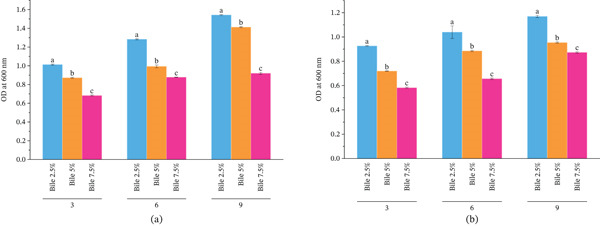
Growth of (a) *W. confusa* OPL2 and (b) *W. cibaria* OPL3 at different bile concentrations (2.5%, 5%, and 7.5%) over time (3, 6, and 9 h). Paired comparison using Tukey′s honestly significant difference (HSD) test showed significant differences among growth. Each bar represents the mean ± standard deviation (*n* = 3). a, b, c indicates the statistically significant difference (*p* < 0.05). Among the LAB strains, *W. cibaria* strain OPL3 grew significantly (*p* < 0.05) higher in 7.5% bile, displaying increased optical density. In contrast, *W. confusa* strain OPL2 was able to grow in a culture medium containing 2.5% bile, but exhibited lower optical density measurements compared with those observed in media containing 5% or 7.5% bile.

The LAB isolates from the intestines of *O. pabda* also demonstrated tolerance to varying concentrations of gut bile (2.5%, 5%, and 7.5%) (Figure 8). The gut bile tolerance test revealed that *W. cibaria* strain OPL3 grew significantly (*p* < 0.05) higher in 7.5% bile, displaying increased OD. In contrast, *W. confusa* strain OPL2 was able to grow in a culture medium containing 2.5% bile, but exhibited lower OD measurements compared with those observed in media containing 5% or 7.5% bile.

#### 3.5.5. Auto‐Aggregation

Both LAB strains showed a significant (*p* < 0.05) increase in autoaggregation over time (Table 6). *W. confusa* OPL2 reached a percentage value of 27.01 ± 1.47, 44.25 ± 0.78, and 64.12 ± 0.53 at the periods of 2, 4, and 6 h, respectively. On the other hand, *W. cibaria* OPL3 appears to have a higher rate of increase (*p* < 0.05), with percentage autoaggregation of 28.77 ± 2.38 at 2 *h* *to* 64.15 ± 0.35 at 6 h.

**Table 6:** Autoaggregation, cell surface hydrophobicity and hemolytic activity of the identified LAB strains.

LAB strains	Time (hours)	Cell surface hydrophobicity (%)		Autoaggregation (%)	Hemolytic activity
		Xylene	Toluene		
*W. confusa* OPL2	2	14.95 ± 5.01^c^	17.69 ± 2.47^c^	27.01 ± 1.47^c^	*γ*
	4	38.47 ± 0.42^b^	43.52 ± 3.04^b^	44.25 ± 0.78^b^	
	6	69.36 ± 0.41^a^	67.73 ± 0.36^a^	64.12 ± 0.53^a^	
*W. cibaria* OPL3	2	15.95 ± 0.65^c^	18.58 ± 2.47^c^	28.77 ± 2.38^c^	*γ*
	4	29.28 ± 3.22^b^	42.87 ± 2.93^b^	40.25 ± 0.51^b^	
	6	68.77 ± 0.17^a^	65.62 ± 0.48^a^	64.15 ± 0.35^a^	

*Note:* Values are shown as means ± standard deviations (*n* = 3). Superscript letters a, b, and c indicate the statistically significant difference (*p* < 0.05) between means.

#### 3.5.6. Coaggregation Ability

**Figure 9:**
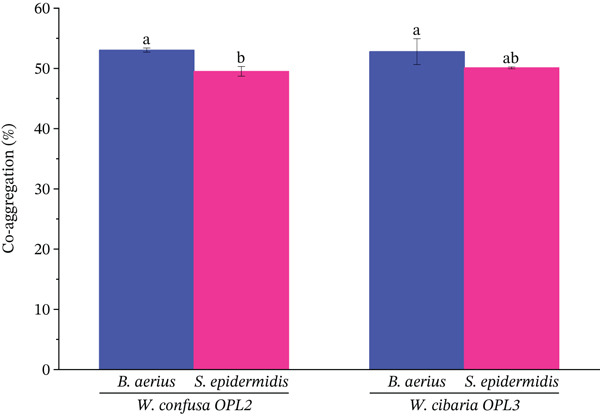
Coaggregation ability of identified LAB strains with two pathogenic bacteria. Each bar represents mean ± SD (*n* = 3); *p* < 0.05. Significant (*p* < 0.05) interaction in the coaggregation assay was observed with the value of 52.79 ± 2.15 for *W. cibaria* OPL3, and 53.05 ± 0.35 for *W. confusa* OPL2 with *B. aerius*, and 50.11 ± 0.16 for *W. cibaria* OPL3, and 49.51 ± 0.81 for *W. confusa* OPL2 with *S. epidermidis*.

Figure 9 showed significant (*p* < 0.05) interaction in the coaggregation assay with the value of 52.79 ± 2.15 for *W. cibaria* OPL3, and 53.05 ± 0.35 for *W. confusa* OPL2 with *B. aerius*, and 50.11 ± 0.16 for *W. cibaria* OPL3, and 49.51 ± 0.81 for *W. confusa* OPL2 with *S. epidermidis* (Figure 9).

#### 3.5.7. Cell Surface Hydrophobicity

Cell surface hydrophobicity of two tested LAB strains increased significantly (*p* < 0.05) with incubation time (Table 6), indicating progressive changes in cell surface properties. At 2 h of incubation, both strains displayed lower hydrophobicity (< 20%) in both solvents, whereas they exhibited the highest hydrophobicity (> 65%) at 6 h, with *W. confusa* OPL2 showing 69.36 ± 0.41 in xylene and 67.73 ± 0.36 in toluene, whereas *W. cibaria* OPL3 displayed 68.77 ± 0.17 in xylene and 65.62 ± 0.48 in toluene.

#### 3.5.8. Hemolytic Activity

Hemolytic activity of the identified LAB strains showed no sign of hemolysis when grown on blood agar plates, indicating *γ*‐hemolysis in nature (Table 6) and therefore safe for host organisms.

#### 3.5.9. Antimicrobial Activity

The antibacterial activity between *W. confusa* OPL2 and *W. cibaria* OPL3 showed that both LAB strains were capable of inhibiting the growth of pathogenic bacteria, including *A. veronii* OPP8 and *P. shigelloides* OPP9 (Table 7).

**Table 7:** Antimicrobial activity of probiotic LAB strains measured as inhibition zone (mm).

Pathogens	Media and incubation temperature	Zone of inhibition (mm)	
		*W. Confusa* OPL2	*W. Cibaria* OPL3
*A. veronii* OPP8	TSA at 37°C	12.9 ± 0.2^a^	12.367 ± 1.021^ab^
*P. shigelloides* OPP9	TSA at 37°C	12.8 ± 0.36^a^	10.9 ± 0.7^b^

*Note:* Values shown are means ± standard deviations (*n* = 3), the superscript letters a and b indicate statistically significant difference (*p* < 0.05).

## 4. Discussion

Gut‐derived LAB offer distinct advantages over many commercial probiotics due to their natural adaptation to the host gut environment, superior colonization capacity, and enhanced physiological resilience [16, 22]. Additionally, their capacity to thrive in host‐specific environments enables stable persistence, heightened ecological fitness, and improved functional performance of probiotics [46, 47]. In this context, the current research imparts novel insights into the intestinal microbiota of *O. pabda* by identifying LAB through morphological, biochemical, and molecular characterization and underscores the in vitro probiotic potentials for exploring as a host‐specific probiotic candidate in sustainable aquaculture.

Building on this rationale, 12 isolates were preliminary screened from the gut content of *O. pabda* for phenotypic analysis. Among these, five bacterial samples exhibited morphological attributes similar to that of typical LAB species. Specifically, these presumptive strains were Gram‐positive, rod‐shaped, and nonmotile. Three displayed facultative anaerobic characteristics in oxygen requirement tests. These attributes align with previous documentation on the probiotic bacteria used in aquaculture, including *Bacillus*, *Lactobacillus*, *Lactococcus*, and *Weissella* that were Gram‐positive, nonmotile, and facultatively anaerobic [15]. In terms of colony characteristics, the majority of the isolates showed circular colonies on agar, white to off‐white pigmentation, smooth edges, and flat to convex elevations. These are the species‐specific attributes of LAB [44, 48].

Biochemical tests further corroborated the results obtained from the morphological analysis. The presumptive LAB isolates OLP2 and OPL3 performed negative results in catalase, indole, and Voges–Proskauer test while exhibiting a positive reaction to methyl red test and acid formation analysis. These findings align with the results reported by Salam et al. [49], Agustina et al. [45], and Shahid et al. [50], who also stated similar results. In addition, the isolates showed positive results in carbohydrate fermentation for sucrose, lactose, maltose, and dextrose except mannitol, indicating their ability to utilize various sugars, a key characteristic of LAB. During sugar fermentation, the production of lactic acid bacteria assures that the isolates are active in acid production, which is an essential attribute of LAB [51, 52]. After biochemical screening, five presumptive LAB isolates were selected for molecular analysis because they consistently matched with typical LAB species.

Although morphological and biochemical characteristics are useful for the preliminary classification of lactic acid bacteria, these approaches alone may lack accuracy and reproducibility when compared with molecular identification techniques [53]. Accordingly, the present study employed an initial phenotypic screening, which was subsequently validated using molecular methods, particularly 16S rRNA gene sequencing to ensure reliable identification of LAB isolates. Finally, molecular analysis confirmed two strains of lactic acid bacteria, that is, *Weissella cibaria* OPL3 and *W. confusa* OPL2 among the five isolates sequenced. The genetic homology between the isolated strains and reference LAB strains suggests that *O. pabda* is a good source of LAB. The results of the molecular analysis aligned with those of biochemical tests, further supporting the reliability of the identification process. Several studies have reported LAB from the gut content of indigenous catfish, including *H. fossilis* [25], *M. cavasius* [24], *Clarias batrachus* [54], *Clarius gariepinus,* [55] and so on. However, earlier studies on *O. pabda* have largely been focused on identifying pathogenic bacteria [56], investigating bacterial community [54], and analyzing postharvest bacterial load [57], with no prior reports on gut‐derived LAB characterization. Therefore, this study provides the first definitive identification of LAB, particularly the genus *Weissella*, from the gastrointestinal tract of *O. pabda*, and decisively addresses a critical gap in current research on this species.

*Weissella* sp. are found to be distributed over various fermented food, sugar cane, cheese and gastrointestinal tract of animals [58–60], as well as aquatic species, including *O. niloticus* [61], *Misgurnus anguillicaudatus* [62], *Siniperca chuatsi* [63], *Onchorhynchs mykiss* [64], *Rastrelliger kanagurta* [65], and *Anguilla bicolor* [66]. This species is recognized as strong probiotic candidate because of its ability to synthesize beneficial enzymes, suppress opportunistic microbes, and release antimicrobial compounds that suppress pathogen proliferation [23, 61, 62, 67]. To argue the statement, several study reported the evidence that the inclusion of *W. cibaria* and *W. confusa* with feed supplements generate measurable benefits in a diverse range of aquatic organisms, including *Onchorhyncus mykiss* [68], *Huso huso* [69], *Acipenser baerii* [70], *O. niloticus* [61], *Carassiu auratus* [71], *S. chuatsi* [63], and *M. anguillicaudatus* [62]. Besides, the applicability of *Weissella* sp. is not confined to finfish, notable effects are also documented in shellfish, such as *M. rosenbergii* [72], *Penaeus vannamei* [23], and *Litopenaeus vannamei* [73], underscoring its versatile potential for aquaculture applications.

Therefore, the identified LAB strains, *W. confusa* OPL2 and *W. cibaria* OPL3, were evaluated for their probiotic potential. Several tests, including pH tolerance (pH 2, pH 3, and pH 5), tolerance to gut bile percentage (2.5%, 5% and 7.5%), growth at various NaCl concentrations (3%, 6%), and thermal conditions (4°C–45°C), were performed. Tolerance to different temperatures and salt concentrations is crucial for LAB to act as probiotics. In the present study, both the identified LAB strains were found to withstand a wide range of temperatures, though the optimum growth was observed at 25°C–37°C, which aligns with the findings of Zhang et al. [74]. The bacteria also grew at low (4°C) and high (45°C) temperatures, with lower proliferation, suggesting that they can survive at both refrigerated and elevated temperatures. The salt tolerance test also revealed that both strains demonstrated high resilience to 3% and 6% NaCl concentrations, which is similar to the findings of Agustina et al. [45]. These findings support the strong probiotic potential of the identified LAB strains.

Another essential criterion to consider LAB as probiotics is the tolerance to the acidic condition [62] and gut bile percentage [45], where they can successfully colonize to benefit the gut microbiota in the host body [75, 76]. In pH tolerance test, *W. confusa* OPL2 and *W. cibaria* OPL3 exhibited strong resistance against pH 2 and pH 3 though higher absorbance was measured at pH 5, indicating their potential survival through the acidic environment of the stomach. Furthermore, the results from bile tolerance test demonstrated that both LAB strains showed remarkable tolerance at gut bile concentrations of 2.5%, 5%, and 7.5%, where *W. cibaria* OPL3 exhibited much higher tolerance with elevated optical absorbance in broth containing 7.5% bile. *W. confusa* OPL2 was able to grow in 2.5% bile with lower optical absorbance than those obtained from culture broth containing 5% and 7.5% bile. The findings are consistent with the results demonstrated by Itoi et al. [77], Zhang et al. [74], and Agustina et al. [45], which suggest their potential features as probiotics.

Colonization of intestinal epithelium is a key functional trait of the probiotic bacteria, governed by physicochemical properties at the cell surface, markedly aggregation capability, and cell surface hydrophobicity [78]. In practical terms, high hydrophobicity enhances the probability of probiotic cells to persist within the dynamic gastrointestinal environment [79]. Hydrophobicity is a critical attribute of cell surfaces that is closely related to the adhesion capacity of probiotic bacteria to epithelial cells. In the present study, both LAB strains displayed proliferated cell‐surface hydrophobicity in different time intervals, reporting the highest rate of 69.36*%* ± 0.41*%* in xylene, and 67.73*%* ± 0.36*%* in toluene. The consistently high values across two hydrophobic solvents reinforce strong hydrophobic surface architecture and align with previously documented reports by Onur and Önlü [80], and Nath et al. [81]. Beyond hydrophobicity, the strains also showed a higher autoaggregation rate at the end of 6 h, ranging from 28.77*%* ± 2.38*%* to 64.15*%* ± 0.35*%* (*W. cibaria* OPL3) and 27.01*%* ± 1.47*%* to 64.12*%* ± 0.53*%* (*W. confusa* OPL2), aligning with the report documented by Mondal et al. [24]. The findings are also consistent with the previous study of LAB, including *Lactococcus lactis* [23], and *Enterococcus* [79]. Autoaggregation supports probiotic function through multiple, mutually reinforcing mechanisms by forming multicellular clusters, facilitating biofilm development that can better withstand gut environmental stress, and serving as a barrier against pathogenic bacteria. Since high autoaggregation values are commonly interpreted as a marker of strong colonization potential, the observed autoaggregation values of *W. confusa* OPL2 and *W. cibaria* OPL3 at approximately 64%, position these strains at the critical threshold that is typically associated with robust gut persistence.

The coaggregation assay is another crucial criterion for the determination of a probiotic candidate. Coaggregation reflects the capacity of a probiotic strain to interact with genetically different pathogenic microorganisms. In this study, the coaggregation rates at the end of 6 h were observed from 52.79 ± 2.15 to 53.05 ± 0.35 (*B. aerius*), and 50.11 ± 0.16 to 49.51 ± 0.81 (*S. epidermidis*) for *W. cibaria* OPL3, and *W. confusa* OPL2, respectively. The finding is comparable with the coaggregation value of *W. confusa* (17%–57%) with *S. aureus* reported by Onur and Önlü [80]. Therefore, the concurrent expression of elevated hydrophobicity, robust autoaggregation, and strong coaggregation properties represents a compelled argument that both LAB strains are well equipped for mucosal establishment that is an essential prerequisite for many probiotic effects.

The antimicrobial activity of probiotic strains is a fundamental characteristic that serves as a natural defense mechanism against pathogenic bacteria by producing substances like organic acids, hydrogen peroxide, bacteriocins or other inhibitory substances [78, 82]. This research utilized two pathogenic bacteria of fish isolated from *O. pabda*, as indicators to determine the antibacterial properties of *Weissella* strains. In the present research, the antimicrobial agents of *W. cibaria* OPL3 showed considerable inhibition zone against *A. veronii* OPP8 and *P. shigelloides* OPP9, exhibiting 12.367 ± 1.021 (mm) and 10.9 ± 0.7 (mm), respectively. Another strain, *W. confusa* OPL2 demonstrated a higher value of inhibition zone against both pathogens, exhibiting 12.9 ± 0.2 (mm) and 12.8 ± 0.36 (mm), respectively. Similar reports were obtained where *Weissella* strains inhibited the growth of pathogenic bacteria, *Bacillus cerius* and *Pseudomonas* by Nath et al. [81].

Antibiotic sensitivity is another important feature of an ideal probiotic strain from the safety key‐point to be applied in fish farming [20]. In the present study, *W. cibaria* OPL3 showed sensitivity against most of the tested antibiotics except for cefotaxime, whereas *W. confusa* OPL2 was found resistant to only erythromycin. This finding is desirable as it reduces the risk of horizontal gene transfer of antibiotic resistance to pathogenic microbes. However, several reports showed antibiotic resistance patterns of probiotic bacteria. For example, *Lactobacillus* sp. showed strong resistance to antibiotics such as kanamycin and vancomycin [80], whereas the percentage of antibiotic resistance of *Weissella* derived from fermented food was found 60% [83]. Therefore, the antibiotic resistance pattern of the identified bacteria in the present study is in line with others suggesting common features of LAB and should be monitored frequently to evaluate the safety standpoint.

The identified LAB strains exhibited no hemolysis zone (*γ* hemolysis) when grown on blood agar for 24 h, ensuring that these strains are nonvirulent in nature, which is a selecting criterion for probiotic strains. Negative results in hemolysis assay have been previously documented in many strains of *Weissella* [84]. The findings suggest that *W. cibaria* OPL3 and *W. confusa* OPL2 strains are safe for animal consumption, which is a critical consideration in selecting probiotic strains.

The overall insight of the experiment states that the autochthonous host‐derived LAB strains from *O. pabda* fish can be considered promising as probiotic candidates in future aquaculture applications, particularly in minimizing gastrointestinal imbalance and improving gut health.

## 5. Conclusion

The study isolated and identified two LAB strains, *W. confusa* OPL2 and *W. cibaria* OPL3 from the intestine of *O. pabda* collected from nearby fish farms and fish markets in the Mymensingh region of Bangladesh. The identified LAB strains exhibited strong in vitro probiotic attributes and were thereby declared as potential probiotic candidates. Future research should be focused on the effective application of these strains in fish farming to improve the overall health condition of fish as well as fish health management.

## Author Contributions

**Rafiatul Jannat Rifa:** concept development, original draft writing, review and editing, experiment methodology, data management, investigation, validation, visualization, formal analysis. **Md. Sakhawat Hossain:** research methodology, formal analysis, review and editing. **Chandrika Mondal:** experimental methodology, review and editing. **Md. Imtiaz Ahamed:** research methodology, review and editing. **Khadiza Tul Kubra:** experimental methodology. **Bushra Bente Rahman Prapti:** research methodology. **Mohammad Matiur Rahman:** concept development, research methodology, supervision, review and editing. **Tanvir Rahman:** research methodology, supervision, resources, review and editing. **Md. Rafiqul Islam Sarder:** supervision, review and editing. **Mariom:** concept development, original draft writing, research methodology, data curation, investigation, formal analysis, validation, visualization, supervision, project administration, resource management, funding acquisition, review and editing.

## Funding

This study was supported by Bangladesh Agricultural University Research System (10.13039/100019278, 2023/49/BAU).

## Disclosure

This manuscript is original, unpublished, and has not been submitted to any other journal. All co‐authors have reviewed the manuscript and consent to its submission.

## Ethics Statement

All applicable rules for the use and care of animals, which might be institutional, national, or international, were followed in this study. The Ethical Standards Research Committee of the Bangladesh Agricultural University Research System (BAURES) in Mymensingh, Bangladesh, approved the research in compliance with the Ethical Standard Protocols (BAURES/ESRC/58/FISH/2024).

## Conflicts of Interest

The authors declare no conflicts of interest.

## Data Availability

All the sequences have been deposited in the NCBI GenBank (https://www.ncbi.nlm.nih.gov/) under the Accession Number SUB15008191.

## References

[1] Goldburg R. and Naylor R. , Future Seascapes, Fishing, and Fish Farming, Frontiers in Ecology and the Environment. (2005) 3, no. 1, 21–28, 10.1890/1540-9295(2005)003[0021:FSFAFF]2.0.CO;2.

[2] Verdegem M. , Buschmann A. H. , Latt U. W. , Dalsgaard A. J. , and Lovatelli A. , The Contribution of Aquaculture Systems to Global Aquaculture Production, Journal of the World Aquaculture Society. (2023) 54, no. 2, 206–250, 10.1111/jwas.12963.

[3] Mariom , Hossain M. S. , Liza M. S. , and Mollah M. F. A. , Optimizing Chicken Blood Quantity for Sustainable Tubificid Worm Cultivation, Journal of Fisheries and Environment. (2025) 49, no. 3, 114–124.

[4] Food and Agriculture Organization , The State of World Fisheries and Aquaculture 2022, 2022, Towards Blue Transformation.

[5] Department of Fisheries (DoF) , Yearbook of Fisheries Statistics of Bangladesh, Fisheries Resources Survey System, 2024, Department of Fisheries, Ministry of Fisheries and Livestock.

[6] Roy D. , Sarker A. K. , Abedin A. M. M. K. , Sarker S. , Begum K. N. , and Latifa G. A. , Some Biological Aspects of Cultured Ompok pabda (Hamilton, 1822) Collected From a Local Fish Farm in Mymensingh, Bangladesh, Aquaculture Studies. (2021) 21, no. 4, 149–159, 10.4194/2618-6381-v21_4_02.

[7] Paul S. , Farjana K. , Mahamud A. S. U. , Mondal D. K. , Tabassum T. , Khoiam M. U. , and Rahman T. , Evaluation of the dietary supplementation of Autochthonous Bacteria on Growth, Survival and Resistance to *Aeromonas veronii* challenge in *Clarias batrachus* and *Heteropneustes fossilis* , Aquaculture, Fish and Fisheries. (2022) 2, no. 5, 364–374, 10.1002/aff2.60.

[8] Fatema K. , Islam M. F. , Sultana N. , and Saha B. , Effects of Supplemented Diets on the Growth Performance and Nutrient Contents of Gulsha tengra, Mystus cavasius, Bangladesh Journal of Zoology. (2017) 45, no. 1, 61–71, 10.3329/bjz.v45i1.34195.

[9] Caipang C. M. A. , Suharman I. , Avillanosa A. L. , and Bargoyo V. T. , Host-Derived Probiotics for Finfish Aquaculture, IOP Conference Series: Earth and Environmental Science (Vol. 430, No 1. p. 012026), 2020, no. 1, IOP Publishing, 10.1088/1755-1315/430/1/012026.

[10] Hasan K. N. and Banerjee G. , Recent Studies on Probiotics as Beneficial Mediator in Aquaculture: A Review, Journal of Basic and Applied Zoology. (2020) 81, no. 1, 10.1186/s41936-020-00190-y.

[11] Hossen S. , Hossain M. S. , Ahamed M. I. , and Mariom , An Analytical Study on Aqua-Chemicals and Drugs Used in Freshwater Aquaculture at Keshabpur Upazila in Jashore, Bangladesh, Critical Insights in Aquaculture. (2026) 2, no. 1, 2646849, 10.1080/29932181.2026.2646849, 2646849.

[12] Ahmad A. , Hassan S. W. , and Banat F. , An Overview of Microalgae Biomass as a Sustainable Aquaculture Feed Ingredient: food Security and Circular Economy, Bioengineered. (2022) 13, no. 4, 9521–9547, 10.1080/21655979.2022.2061148. 35387561 PMC9161971

[13] Lazado C. C. , Caipang C. M. A. , and Estante E. G. , Prospects of Host-Associated Microorganisms in Fish and Penaeids as Probiotics With Immunomodulatory Functions, Fish & Shellfish Immunology. (2015) 45, no. 1, 2–12, 10.1016/j.fsi.2015.02.023, 25703713. 25703713

[14] Mariom , Hossain M. S. , Rifa M. S. , Mondal C. , Ahamed M. I. , and Sudipta A. P. , Lactic Acid Bacteria (LAB) in Aquaculture: Current Insights, Research Gaps, and Future Directions for Sustainability, Archives of Microbiology. (2026) 208, no. 1. 10.1007/s00203-025-04625-4 41396260

[15] Martínez Cruz P. , Ibáñez A. L. , Monroy Hermosillo O. A. , and Ramírez Saad H. C. , Use of Probiotics in Aquaculture, International Scholarly Research Notices. (2012) 2012, no. 1, 916845, 10.5402/2012/916845, 23762761.

[16] Chizhayeva A. , Amangeldi A. , Oleinikova Y. , Alybaeva A. , and Sadanov A. , Lactic Acid Bacteria as Probiotics in Sustainable Development of Aquaculture, Aquatic Living Resources. (2022) 35, 10.1051/alr/2022011.

[17] Munni M. J. , Akther K. R. , Ahmed S. , Hossain M. A. , and Roy N. C. , Effects of Probiotics, Prebiotics, and Synbiotics as an Alternative to Antibiotics on Growth and Blood Profile of Nile Tilapia (*Oreochromis niloticus*), Aquaculture Research. (2023) 2023, no. 1, 12, 2798279, 10.1155/2023/2798279.

[18] Chauhan A. and Singh R. , Probiotics in Aquaculture: A Promising Emerging Alternative Approach, Symbiosis. (2019) 77, no. 2, 99–113, 10.1007/s13199-018-0580-1.

[19] Pereira W. A. , Mendonça C. M. N. , Urquiza A. V. , Marteinsson V. Þ. , LeBlanc J. G. , Cotter P. D. , and Oliveira R. P. , Use of Probiotic Bacteria and Bacteriocins as an Alternative to Antibiotics in Aquaculture, Microorganisms. (2022) 10, no. 9, 10.3390/microorganisms10091705, 36144306. PMC9503917 36144306

[20] Alonso S. , Carmen C. M. , Berdasco M. , de la Banda I. G. , Moreno-Ventas X. , and de Rojas A. H. , Isolation and Partial Characterization of lactic Acid Bacteria From the Gut Microbiota of Marine Fishes for Potential Application as Probiotics in Aquaculture, Probiotics and Antimicrobial Proteins. (2019) 11, no. 2, 569–579, 10.1007/s12602-018-9439-2, 29959637. 29959637

[21] Govindaraj K. , Samayanpaulraj V. , Narayanadoss V. , and Uthandakalaipandian R. , Isolation of Lactic Acid Bacteria From Intestine of Freshwater Fishes and Elucidation of Probiotic Potential for Aquaculture Application, Probiotics and Antimicrobial Proteins. (2021) 13, no. 6, 1598–1610, 10.1007/s12602-021-09811-6, 34164781. 34164781

[22] Naiel M. A. , Farag M. R. , Gewida A. G. , Elnakeeb M. A. , Amer M. S. , and Alagawany M. , Using lactic Acid Bacteria as an Immunostimulants in Cultured Shrimp With Special Reference to *Lactobacillus* spp, Aquaculture International. (2021) 29, no. 1, 219–231, 10.1007/s10499-020-00620-2.

[23] Kanjan P. , Kimtun A. , Chaimongkol S. , and Suanyuk N. , Indigenous ProbioticsWeissella *cibariaandSaccharomyces* cerevisiaeon Growth, Microbial Population, and Survival of Pacific White Shrimp (*Penaeus vannamei*) Challenged WithVibrio parahaemolyticus (AHPND Strains), Aquaculture Research. (2025) 2025, no. 1, 9910236, 10.1155/are/9910236.

[24] Mondal C. , Hossain M. S. , Rifa R. J. , Ahamed M. I. , Tanni T. A. , Hossain Z. , Rahman T. , Siddique M. P. , and Mariom , Isolation and Molecular Characterization of Gut-Derived Lactic Acid Bacteria From the Gangetic Mystus, *Mystus Cavasius* With Promising Probiotic Attributes, Archives of Microbiology. (2026) 208, no. 4, 10.1007/s00203-026-04735-7, 41627457. 41627457

[25] Hossain M. S. , Mondal C. , Rifa R. J. , Ahamed M. I. , Rahman M. M. , Rahman T. , Siddique M. P. , and Mariom , Isolation and Characterization of Lactic Acid Bacteria From *Heteropneustes fossilis* for Probiotic Applications in Aquaculture, Scientific Reports. (2026) 16, no. 1, 5835, 10.1038/s41598-026-35791-0, 41559126. 41559126 PMC12894858

[26] Zhu L. , Kong Y. , Chang X. , Feng J. , Wang X. , Hou L. , and Kong X. , Effects of Two Fish-Derived Probiotics on Growth Performance, Innate Immune Response, intestinal health, and Disease Resistance of *Procambarus clarkii* , Aquaculture. (2023) 562, 738765, 10.1016/j.aquaculture.2022.738765.

[27] Kader M. A. , Zahidah Azahar N. , Iehata S. , Bulbul M. , Islam M. M. , Sarker J. , Mariom , Rahman M. M. , and Asaduzzaman M. , Dietary Supplementation of Host-Associated Lactic Acid Bacteria Modulates Growth, Metabolic Activities, and Immune-Related Gene Expression in Giant Freshwater *prawn,Macrobrachium rosenbergii* , Journal of the World Aquaculture Society. (2021) 52, no. 1, 216–230, 10.1111/jwas.12734.

[28] Zhao C. , Guo G. , Li Z. , Chen J. , and Ren Y. , Effects of Probiotics (*Bacillus coagulans*) Supplementation After Antibiotic Administration on Growth, Immunity, and Intestinal Microflora in Turbot *Scophthalmus maximus* , Aquaculture International. (2024) 32, no. 2, 1473–1491, 10.1007/s10499-023-01225-1.

[29] Pingki M. T. , Hossain M. S. , Hasan M. , Bilasi S. N. , and Rahman M. M. , Ethical Consideration of Wildlife Research in Bangladesh, Bangladesh Journal of Zoology. (2026) 53, no. 3, 389–410, 10.3329/bjz.v53i3.88377.

[30] Schillinger U. and Lücke F. K. , Identification of Lactobacilli From Meat and Meat Products, Food Microbiology. (1987) 4, no. 3, 199–208, 10.1016/0740-0020(87)90002-5.

[31] Dykes G. A. , Britz T. J. , and Holy V. A. , Numerical Taxonomy and Identification of Lactic Acid Bacteria From Spoiled, Vacuum-Packaged Vienna Sausages, Journal of Applied Microbiology. (1994) 76, no. 3, 246–252, 10.1111/j.1365-2672.1994.tb01623.x, 8157544. 8157544

[32] Jain A. , Jain R. , Jain S. , Jain A. , Jain R. , and Jain S. , Motility Testing–Hanging Drop Method and Stab, Basic Techniques in Biochemistry, Microbiology and Molecular Biology: Principles and Techniques, 2020, Springer, 121–122, 10.1007/978-1-4939-9861-6_34.

[33] Reiner K. , Catalase Test Protocol, American Society for Microbiology. (2010) 1, no. 1.

[34] McDevitt S. , Methyl Red and Voges-Proskauer Test Protocols, 2009, American Society for Microbiology.

[35] Müller T. , Comparison of Methods for Differentiation Between Homofermentative and Heterofermentative Lactic Acid Bacteria, Zentralblatt Für Mikrobiologie. (1990) 145, no. 5, 363–366, 10.1016/S0232-4393(11)80064-8.

[36] Bulut C. , Gunes H. , Okuklu B. , Harsa S. , Kilic S. , Coban H. S. , and Yenidunya A. F. , Homofermentative Lactic Acid Bacteria of a Traditional Cheese, Comlek peyniri from Cappadocia Region, Journal of Dairy Research. (2005) 72, no. 1, 19–24, 10.1017/S0022029904000536, 15747727. 15747727

[37] Vergin K. L. , Urbach E. , Stein J. L. , DeLong E. F. , Lanoil B. D. , and Giovannoni S. J. , Screening of a Fosmid Library of Marine Environmental Genomic DNA Fragments Reveals Four Clones Related to Members of the Order *Planctomycetales* , Applied and Environmental Microbiology. (1998) 64, no. 8, 3075–3078, 10.1128/AEM.64.8.3075-3078.1998, 9687477. 9687477 PMC106819

[38] Stephen F. A. , Basic Local Alignment Search tool, Journal of Molecular Biology. (1990) 215, no. 3, 403–410, 10.1016/S0022-2836(05)80360-2. 2231712

[39] Patel P. , Patel B. , Amaresan N. , Joshi B. , Shah R. , and Krishnamurthy R. , Isolation and Characterization of *Lactococcus garvieae* From the Fish Gut for In Vitro Fermentation With Carbohydrates From Agro-Industrial Waste, Biotechnology Reports. (2020) 28, e00555, 10.1016/j.btre.2020.e00555, 33294403. 33294403 PMC7691725

[40] Collado M. C. , Meriluoto J. , and Salminen S. , Adhesion and Aggregation Properties of Probiotic and Pathogen Strains, European Food Research and Technology. (2008) 226, no. 5, 1065–1073, 10.1007/s00217-007-0632-x.

[41] Fonseca H. C. , de Sousa M. D. , Ramos C. L. , Dias D. R. , and Schwan R. F. , Probiotic Properties of Lactobacilli and Their Ability to Inhibit the Adhesion of Enteropathogenic Bacteria to Caco-2 and HT-29 cells, Probiotics and Antimicrobial Proteins. (2021) 13, no. 1, 102–112, 10.1007/s12602-020-09659-2, 32415515. 32415515

[42] Yasmin I. , Saeed M. , Khan W. A. , Khaliq A. , Chughtai M. F. J. , Iqbal R. , and Tanweer S. , In Vitro Probiotic Potential and Safety Evaluation (Hemolytic, Cytotoxic Activity) of Bifidobacterium Strains Isolated From Raw Camel Milk, Microorganisms. (2020) 8, no. 3, 10.3390/microorganisms8030354, 32131456. PMC7143641 32131456

[43] Petjul K. , Khunsanit P. , Kollboon U. , and Boonmee T. , Evaluation of Enzymatic Activity, Hemolytic Safety, and Antibiotic Susceptibility of Bacillus spp. Isolated From Giant Freshwater Prawn (Macrobrachium rosenbergii) Farms in Kalasin Province, Thailand, Veterinary World. (2025) 18, no. 11, 3622–3630, 10.14202/vetworld.2025.3622-3630, 41472778. 41472778 PMC12745024

[44] Thi Q. V. C. , Dung T. Q. , Hien H. N. , Trung N. B. , Dung T. T. , and Thuy N. P. , Antibacterial Activity of Lactic Acid Bacteria From Various Freshwater Fish Species Against Pathogenic Bacteria in Caged Red Tilapia (*Oreochromis* sp.), Biodiversitas Journal of Biological Diversity. (2023) 24, no. 6, 10.13057/biodiv/d240633.

[45] Agustina A. , Saptiani G. , and Hardi E. H. , Isolation and Identification of Potential Lactic Acid Bacteria as Probiotics From the Intestines of Repang Fish (*Puntioplites waandersi*), Aquaculture, Aquarium, Conservation & Legislation. (2022) 15, no. 1, 24–33.

[46] Ringø E. and Gatesoupe F. J. , Lactic Acid Bacteria in Fish: a Review, Aquaculture. (1998) 160, no. 3-4, 177–203, 10.1016/S0044-8486(97)00299-8.

[47] Mohapatra S. , Chakraborty T. , Prusty A. K. , Das P. , Paniprasad K. , and Mohanta K. N. , Use of Different Microbial Probiotics in the Diet of rohu, *Labeo rohita* Fingerlings: Effects on Growth, Nutrient Digestibility and Retention, Digestive Enzyme Activities and Intestinal Microflora, Aquaculture Nutrition. (2012) 18, no. 1, 1–11, 10.1111/j.1365-2095.2011.00866.x.

[48] Vázquez J. A. , González M. , and Murado M. A. , Effects of Lactic Acid Bacteria Cultures on Pathogenic Microbiota From Fish, Aquaculture. (2005) 245, no. 1-4, 149–161, 10.1016/j.aquaculture.2004.12.008.

[49] Salam M. A. , Islam M. A. , Paul S. I. , Rahman M. M. , Rahman M. L. , Islam F. , and Islam T. , Gut Probiotic Bacteria of *Barbonymus gonionotus* Improve Growth, Hematological Parameters and Reproductive Performances of the Host, Scientific Reports. (2021) 11, no. 1, 10692, 10.1038/s41598-021-90158-x, 34021215. 34021215 PMC8140159

[50] Shahid M. , Hussain B. , Riaz D. , Khurshid M. , Ismail M. , and Tariq M. , Identification and Partial Characterization of Potential Probiotic Lactic acid Bacteria in freshwaterLabeo *rohitaandCirrhinus mrigala* , Aquaculture Research. (2017) 48, no. 4, 1688–1698, 10.1111/are.13006.

[51] Holzapfel W. H. , Haberer P. , Geisen R. , Björkroth J. , and Schillinger U. , Taxonomy and Important Features of Probiotic Microorganisms in Food and Nutrition, American Journal of Clinical Nutrition. (2001) 73, no. 2, 365s–373s, 10.1093/ajcn/73.2.365s, 11157343. 11157343

[52] Hakkinen M. , Faqih A. R. , Prihanto A. A. , and Anitasari S. , Isolation of Lactic Acid Bacteria as Potential Probiotic Candidates From the Digestive Tract of *Gobiopterus* sp, Biodiversitas Journal of Biological Diversity. (2025) 26, no. 2, 1008–1017, 10.13057/biodiv/d260249.

[53] Balcázar J. L. , De Blas I. , Ruiz-Zarzuela I. , Vendrell D. , Girones O. , and Muzquiz J. L. , Sequencing of Variable Regions of the 16S rRNA Gene for Identification of Lactic Acid bacteria Isolated From the Intestinal Microbiota of Healthy Salmonids, Comparative Immunology, Microbiology and Infectious Diseases. (2007) 30, no. 2, 111–118, 10.1016/j.cimid.2006.12.001, 17239438. 17239438

[54] Ganguly A. , Banerjee A. , Mandal A. , Khan M. A. , and Mohapatra P. K. D. , Isolation and Characterization of Bacteria From the Intestine of *Clarias batrachus* for Probiotic Organism, Proceedings of the Zoological Society (Vol. 72, No. 4, pp. 411-419), 2019, Springer, 10.1007/s12595-018-0283-x.

[55] Nurhayati A. P. , Zulaika E. , Amin M. , Setiawan E. , and Wijaya Z. M. , Isolation and Screening of Lactic Acid Bacteria producinganti-Edwardsiellafrom the Gastrointestinal Tract of Wild Catfish (*Clarias gariepinus*) for Probiotic Candidates, Open Agriculture. (2023) 8, no. 1, 20220212, 10.1515/opag-2022-0212.

[56] Simu Z. A. , Masuma U. , Farjana K. , Mahamud A. S. U. , Rahman T. , and Chowdhury M. B. R. , Investigation of a Bacterial Pathogen Isolated From Farmed *Ompok pabda* , Bangladesh Journal of Fisheries. (2019) 31, no. 2, 243–252.

[57] Akter T. and Chowdhury G. W. , Post harvest Bacterial Load in the Gut of *Ompok pabda* (Hamilton, 1822) From Two Fish Markets of Brahmanbaria, Bangladesh Journal of Zoology. (2019) 47, no. 2, 243–251, 10.3329/bjz.v47i2.44335.

[58] Fusco V. , Chieffi D. , Fanelli F. , Montemurro M. , Rizzello C. G. , and Franz C. M. , The Weissella and Periweissella genera: Up-to-Date Taxonomy, Ecology, Safety, Biotechnological, and Probiotic Potential, Frontiers in Microbiology. (2023) 14, 1289937, 10.3389/fmicb.2023.1289937, 38169702. 38169702 PMC10758620

[59] Ahn S. B. , Park H. E. , Lee S. M. , Kim S. Y. , Shon M. Y. , and Lee W. K. , Characteristics and Immuno-Modulatory Effects of *Weissella cibaria* JW15 Isolated From Kimchi, Korea Traditional Fermented Food, for Probiotic Use, Journal of Biomedical Research. (2013) 14, no. 4, 206–211, 10.12729/jbr.2013.14.4.206.

[60] Le B. and Yang S. H. , Isolation of *Weissella* Strains as Potent Probiotics to Improve Antioxidant Activity of Salted Squid by Fermentation, Journal of Applied Biological Chemistry. (2018) 61, no. 1, 93–100, 10.3839/jabc.2018.014.

[61] Sreelakshmi K. , Kadirvelu K. , and Ramasubramanian V. , Probiotic Efficacy, Antioxidant, Antimicrobial and Anticancer Activities of Cell Free Supernatant Derived From *Weissella confusa* Isolated From *Oreochromis niloticus*–An In Vitro Study, Microbe. (2025) 6, 100291, 10.1016/j.microb.2025.100291.

[62] Yang B. , Song H. , Hu R. , Tao L. , Liang Z. , Cong W. , and Kang Y. , Weissella confusa N17 Derived From Loach (Misgurnus anguillicaudatus) Exhibits Promising for Further Applications in Loach Aquaculture, Probiotics and Antimicrobial Proteins. (2025) 17, no. 1, 212–226, 10.1007/s12602-023-10149-4, 37632675. 37632675

[63] Wang J. , Hao Y. , Zhang L. , Gao X. , Xu Y. , Wang J. , Hanafiah F. , Khor W. , Sun Y. , and Wu C. , Profiling the Gut Structure and Microbiota, and Identifying Two Dominant Bacteria Belonging to the *Weissella* Genus in Mandarin fish (*Siniperca chuatsi*) Fed an Artificial Diet, Frontiers in Microbiology. (2024) 15, 1486501, 10.3389/fmicb.2024.1486501, 39678912. 39678912 PMC11639983

[64] Mortezaei F. , Royan M. , Allaf Noveirian H. , Babakhani A. , Alaie Kordghashlaghi H. , and Balcázar J. L. , In Vitro Assessment of Potential Probiotic Characteristics of Indigenous *Lactococcus lactis* and *Weissella oryzae* Isolates From Rainbow Trout (*Oncorhynchus mykiss Walbaum*), Journal of Applied Microbiology. (2020) 129, no. 4, 1004–1019, 10.1111/jam.14652, 32248610. 32248610

[65] Ardyati T. and Jatmiko Y. D. , Selection of Potential Lactic Acid Bacteria From Fish Intestine of Mackerel (*Rastrelliger kanagurta*) From Lembata Regency, East Nusa Tenggara, Indonesia. Malaysian, Journal of Microbiology. (2022) 18, 10.21161/mjm.211302.

[66] Dinoto A. , Sulistiani S. , Handayani R. , and Julistiono H. , *Weissella paramesenteroides* From Intestine of Indonesian Eel (*Anguilla bicolor* McClelland) and Their Potential Antimicrobial Property, AIP Conference Proceedings (Vol. 2021, No. 1, p. 070001), 2018, AIP Publishing LLC, 10.1063/1.5062799.

[67] Quintanilla-Pineda M. , Ibañez F. C. , Garrote-Achou C. , and Marzo F. , A Novel Postbiotic Product Based on *Weissella cibaria* for Enhancing Disease Resistance in Rainbow Trout: Aquaculture Application, Animals. (2024) 14, no. 5, 10.3390/ani14050744, 38473129. PMC10930836 38473129

[68] Kahyani F. , Pirali-Kheirabadi E. , Shafiei S. , and Shenavar Masouleh A. , Effect of Dietary Supplementation of Potential probioticWeissella confusaon Innate Immunity, Immune-Related Genes Expression, Intestinal Microbiota and Growth Performance of Rainbow Trout (*Oncorhynchus mykiss*), Aquaculture Nutrition. (2021) 27, no. 5, 1411–1420, 10.1111/anu.13279.

[69] Yeganeh Rastekenari H. , Kazami R. , Shenavar Masouleh A. , Banavreh A. , Najjar Lashgari S. , Sayed Hassani M. H. , and Hallajian A. , Autochthonous probioticsLactococcus *lactisandWeissella* confusain the Diet of Fingerlings Great *sturgeon,Huso huso*: Effects on Growth Performance, Feed Efficiency, Haematological Parameters, Immune Status and Intestinal Morphology, Aquaculture Research. (2021) 52, no. 8, 3687–3695, 10.1111/are.15213.

[70] Hashemimofrad M. , Sattari M. , Khoshkholgh M. , Shenavar Masuoleh A. , and Abasalizadeh A. , Effect of *Weissella cibaria* as Probiotic on Some on Growth Factors in Siberian Sturgeon *Acipenser baerii* , Iranian Scientific Fisheries Journal. (2016) 25, no. 2, 17–27.

[71] Zhu X. K. , Yang B. T. , Hao Z. P. , Li H. Z. , Cong W. , and Kang Y. H. , Dietary Supplementation With *Weissella cibaria* C-10 and Bacillus Amyloliquefaciens T-5 Enhance Immunity Against *Aeromonas veronii* Infection in Crucian Carp (*Carassiu auratus*), Microbial Pathogenesis. (2022) 167, 105559, 10.1016/j.micpath.2022.105559, 35568093. 35568093

[72] Tardecilla K. M. C. and Maningas M. B. B. , Evaluation of Inhibitory, Immunomodulatory, Survival, and Growth Effects of Host-Derived *Weissella confusa* on *Macrobrachium rosenbergii* Challenged with *Vibrio parahaemolyticus* , Fish & Shellfish Immunology. (2024) 154, 109964, 10.1016/j.fsi.2024.109964, 39401740. 39401740

[73] Huy N. D. , Ngoc L. M. T. , Loc N. H. , Lan T. T. , Quang H. T. , and Dung T. , Isolation of *Weissella cibaria* From Pacific White Shrimp (*Litopenaeus vannamei*) Gastrointestinal Tract and Evaluation of Its Pathogenic Bacterial Inhibition, Indian Journal of Science & Technology. (2020) 13, no. 10, 1200–1212, 10.17485/ijst/2020/v13i10/149934.

[74] Zhang W. , Lai S. , Zhou Z. , Yang J. , Liu H. , Zhong Z. , and Peng G. , Screening and Evaluation of Lactic Acid Bacteria With Probiotic Potential From Local Holstein Raw Milk, Frontiers in Microbiology. (2022) 13, 918774, 10.3389/fmicb.2022.918774, 35979483. 35979483 PMC9377552

[75] Akinyemi M. O. , Ogunremi O. R. , Adeleke R. A. , and Ezekiel C. N. , Probiotic Potentials of Lactic Acid Bacteria and Yeasts From Raw Goat Milk in Nigeria, Probiotics and Antimicrobial Proteins. (2024) 16, no. 1, 163–180, 10.1007/s12602-022-10022-w, 36520357. 36520357

[76] Marchwińska K. and Gwiazdowska D. , Isolation and Probiotic Potential of Lactic Acid Bacteria From Swine Feces for Feed Additive Composition, Archives of Microbiology. (2022) 204, no. 1, 10.1007/s00203-021-02700-0, 34940898. PMC8702511 34940898

[77] Itoi S. , Abe T. , Washio S. , Ikuno E. , Kanomata Y. , and Sugita H. , Isolation of Halotolerant *Lactococcus lactis* subsp. *lactis* From Intestinal Tract of Coastal Fish, International Journal of Food Microbiology. (2008) 121, no. 1, 116–121, 10.1016/j.ijfoodmicro.2007.11.031, 18068256. 18068256

[78] Moradi M. , Mardani K. , and Tajik H. , Characterization and Application of postbiotics of *Lactobacillus* spp. on *Listeria monocytogenes* In Vitro and in Food Models, LWT. (2019) 111, 457–464, 10.1016/j.lwt.2019.05.072.

[79] Ayyash M. , Abushelaibi A. , Al-Mahadin S. , Enan M. , El-Tarabily K. , and Shah N. , In-vitro Investigation Into Probiotic Characterisation of *Streptococcus* and *Enterococcus* Isolated From Camel Milk, LWT. (2018) 87, 478–487, 10.1016/j.lwt.2017.09.019.

[80] Onur M. and Önlü H. , Isolation, Characterization of *Weissella confusa* and *Lactococcus lactis* From Different Milk Sources and Determination of Probiotic Features, Brazilian Journal of Microbiology. (2024) 55, no. 1, 663–679, 10.1007/s42770-023-01208-7, 38158467. 38158467 PMC10920558

[81] Nath S. , Roy M. , Sikidar J. , Deb B. , Sharma I. , and Guha A. , Characterization and In-Vitro Screening of Probiotic Potential of Novel *Weissella confusa* Strain GCC_19R1 Isolated From Fermented Sour Rice, Current Research in Biotechnology. (2021) 3, 99–108, 10.1016/j.crbiot.2021.04.001.

[82] Rajakovich L. J. and Balskus E. P. , Metabolic Functions of the Human Gut Microbiota: The Role of Metalloenzymes, Natural Product Reports. (2019) 36, no. 4, 593–625, 10.1039/C8NP00074C. 30452039 PMC7771511

[83] Muñoz-Atienza E. , Gómez-Sala B. , Araújo C. , Campanero C. , Del Campo R. , Hernández P. E. , and Cintas L. M. , Antimicrobial Activity, Antibiotic Susceptibility and Virulence Factors of Lactic Acid Bacteria of Aquatic Origin Intended for Use as Probiotics in Aquaculture, BMC Microbiology. (2013) 13, 10.1186/1471-2180-13-15, 23347637. PMC3574848 23347637

[84] Sharma S. , Kandasamy S. , Kavitake D. , and Shetty P. H. , Probiotic Characterization and Antioxidant Properties of *Weissella confusa* KR780676, Isolated From an Indian Fermented Food, LWT. (2018) 97, 53–60, 10.1016/j.lwt.2018.06.033.

